# Discovery of curcumin inspired sulfonamide derivatives as a new class of carbonic anhydrase isoforms I, II, IX, and XII inhibitors

**DOI:** 10.1080/14756366.2017.1380638

**Published:** 2017-10-02

**Authors:** P. V. Sri Ramya, Srinivas Angapelly, Andrea Angeli, Chander Singh Digwal, Mohammed Arifuddin, Bathini Nagendra Babu, Claudiu T. Supuran, Ahmed Kamal

**Affiliations:** a Department of Medicinal Chemistry, National Institute of Pharmaceutical Education and Research (NIPER), Hyderabad, India;; b NEUROFARBA Department, Università degli Studi di Firenze, Sezione di Scienze Farmaceutiche, Florence, Italy

**Keywords:** Carbonic anhydrase, isoforms I, II, IX and XII, sulfonamide, curcumin

## Abstract

A series of curcumin inspired sulfonamide derivatives was prepared from various chalcones and 4-sulfamoyl benzaldehyde *via* Claisen–Schmidt condensation. All new compounds were assayed as inhibitors of four human isoforms of the metalloenzyme carbonic anhydrase (hCA, EC 4.2.1.1) isoforms hCA I, II, IX and XII. Interesting inhibitory activities were observed against all these isoforms. hCA I, an isoform involved in several eye diseases was inhibited moderately with K_I_s in the range of 191.8–904.2 nM, hCA II, an antiglaucoma drug target was highly inhibited by the new sulfonamides, with K_I_s in the range of 0.75–8.8 nM. hCA IX, a tumor-associated isoform involved in cancer progression and metastatic spread was potently inhibited by the new sulfonamides, with K_I_s in the range of 2.3–87.3 nM, whereas hCA XII, and antiglaucoma and anticancer drug target, was inhibited with K_I_s in the range of 6.1–71.8 nM. It is noteworthy that one of the new compounds, **5d**, was found to be almost 9 times more selective against hCA II (K_I_ = 0.89 nM) over hCA IX and hCA XII, whereas **5e** was 3 and 70 times more selective against hCA II (K_I_ = 0.75 nM) over hCA IX and hCA XII, respectively.

## Introduction

Carbonic anhydrase (CAs, EC 4.2.1.1) enzymes are ubiquitous metalloproteins present in prokaryotes and eukaryotes, and they catalyze the fundamental biochemical process of carbon dioxide hydration (a reversible reaction producing a bicarbonate anion and a proton), being therefore one of the principal regulators of cellular pH homeostasis^1^. These enzymes also take part in several vital biological processes such as carbon dioxide and bicarbonate ion transport, respiration, electrolyte secretion, bone resorption, gluconeogenesis, lipogenesis and ureagenesis among others^2–6^. Indeed, seven (α-, β-, γ-, δ-, ζ-, η-, and θ-) genetically different CA families are known so far, which incorporate different metal ions at their active site including, Zn(II) (in all classes), Fe(II) (in the γ-CAs), Co(II) (in δ-CAs), and Cd(II) (in ζ-CAs)^7^^,^^8^. To date, in mammals, 15 α-CA isozymes have been identified, that differ in subcellular localization, tissue distribution, and catalytic activity^1^^,^^2^. Some of these isozymes are cytosolic (CA I, CA II, CA III, CA VII, and CA XIII), others are membrane bound (CA IV, CA IX, CA XII, and CA XIV), two are mitochondrial (CA VA and CA VB), and one is secreted in saliva and milk (CA VI)^2^^,^^9–12^.

**Figure 1.**
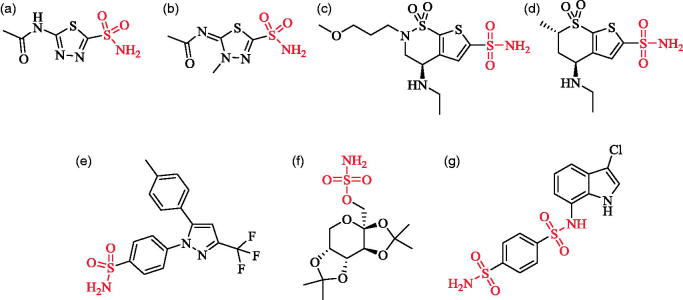
Structures of some drugs and drug candidates possessing sulfonamide/sulfamate moiety.

The potential of this enzyme family as an important class of biological targets for pharmacologic intervention was recognized several decades ago^13^. An atypical expression level or dysregulated activities of these enzymes were proven to be linked with several human diseases, such as glaucoma (hCAs I, II, IV, and XII), cancer (hCAs IX and XII), some central-nervous system syndromes, including epilepsy, neuropathic pain, and idiopathic intracranial hypertension (hCAs I, II, and VII), edema (hCA II, IV, XII, and XIV), obesity (hCAs VA and VB), and osteoporosis (hCA IV and XIV)^9^^,^^11^^,^^14^^,^^15^. Currently, the known carbonic anhydrase inhibitors (CAIs) can be divided into various classes: those that coordinate to the active site metal ion^9^ and those that do not interact with it^16^. Primary sulfonamides are the central and historically most prominent class of CAIs, being discovered in 1940, with many representatives in clinical use for decades^17^. The sulfonamide functionality, denoted as a zinc-binding group (ZBG), coordinates in deprotonated form to the Zn(II) ion within the hCA active sites and establishes hydrogen bonds with a residues nearby (e.g. Thr199 in α-CAs)^3^^,^^4^. Such binding features are common among the active site architectures of all the 15 human isozymes, that all belong to the α-class^9^^,^^14^. Furthermore, mercaptophenols, ureates/hydroxamates, metal complexing anion inhibitors, and the bioisosteres of sulfonamides (such as sulfamates and sulfamides) exhibit CA inhibitory activity following a similar mechanism^9^^,^^16^. Some of the structures of CAIs [acetazolamide, methazolamide, brinzolamide, dorzolamide (antiglaucoma drugs), celecoxib (COX-2 selective nonsteroidal anti-inflammatory drug), topiramate (anticonvulsant drug), and indisulam (in clinical development as an anticancer agent)] in clinical use/development are depicted in Figure 1. The main drawback associated with the use of CAIs is their lack of selectivity in inhibiting various isoforms, considering the fact that many of these isoforms are rather similar from the structural view point and even subcellular localization^3^^,^^4^^,^^9^, resulting thus in undesired side effects^3^^,^^4^. Thus, it is still challenging to design selective/specific agents with distinct inhibition profiles (inhibitors or activators) for any of these isoforms. In the last decades, many efforts have been carried out to design isoform-selective sulfonamide-inhibitors by employing two principal methods: the ring and the tail approaches^9^^,^^14^^,^^18^^,^^19^. The first resides in modulating the ring (chiefly its chemical nature) directly connected to the sulfonamide ZBG, whereas the latter consists in attaching different tails to the aromatic/heterocyclic ring carrying various ZBGs, of the sulfonamide, sulfamide, sulfamate, carboxylate, hydroxamate, or dithiocarbamate type^3^^,^^4^. This enabled modulation of the interactions that the ligand establishes with the middle and outer parts of the active site cavity, which are the most variable regions among the 15 hCA isoforms mentioned above, and led to a variety of isoform-selective CAIs^3^^,^^4^^,^^9^.

Among the CA isoforms, CA I and CA II are the two major isozymes present at high concentrations in the cytosol in erythrocytes, and CA II (together with CA IX) is the most active among all the α-CAs^2^^,^^9–12^. Sulfonamides which are known as strong inhibitors of CA II, have been utilized as commercial drugs (Figure 1, structures A, B, C, and D) to treat glaucoma or diuretics for a long period^20^. In view of lack of selectivity and side effects associated with the use of the existing drugs, exhaustive search for novel CAIs is ongoing either through synthesis of new derivatives of known drugs or from new molecular bases.

**Figure 2.**
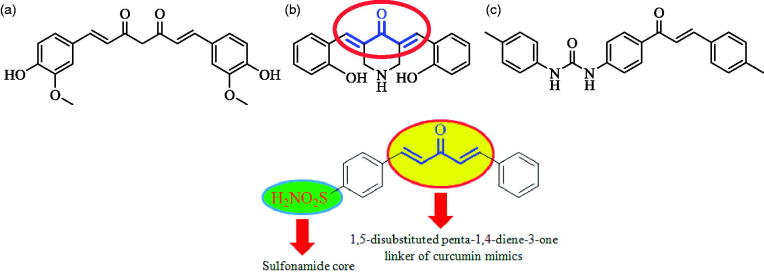
Design of curcumin inspired sulfonamide derivatives as hCA inhibitors.

Systematic search for new chemotypes may be tackled by investigating compounds from natural products^21^^,^^22^. Natural compounds such as resveratrol, catechin, silymarin, dobutamin, and curcumin (K_I_ = 7.44 µM against hCA II isozyme) exhibit CA inhibitory activity^23^. Curcumin, the main curcuminoid of popular Indian spice turmeric (*Curcuma longa*), was reported to possess neuroprotective properties, which may be effective in the prevention and treatment of glaucoma^24^. Curcumin as well as its analogues and 4′-(phenylurenyl)chalcones (Figure 2) were proven to act as CAIs^25^^,^^26^. Intrigued by these findings and in continuation of our research into the synthesis and biological evaluation of curcumin inspired analogues^27^, herein, we wish to report curcumin inspired sulfonamide derivatives as a new class of CAIs against isoforms I, II, IX, and XII.

## Materials and methods

### Chemistry

All reagents and solvents were obtained from commercial suppliers and used without further purification. The reactions were monitored by thin layer chromatography (TLC), using MERCK pre-coated silica gel 60-F_254_ aluminum plates. Column chromatography with 60–120 mesh silica gel was used as separation and purification method. Ethyl acetate and hexane were used as eluents. Melting points were obtained on Stuart digital melting-point apparatus/SMP 30 and were uncorrected. All IR spectra were recorded on a Perkin Elmer, FT-IR spectrometer using KBr discs. ^1^H NMR spectra were recorded on an Avance NMR instrument operated at 500 MHz. ^13^C NMR spectra were recorded on an Avance NMR instrument operated at 125 MHz. Chemical shift values were reported in ppm with TMS as an internal reference and *J* values were given in Hertz. The following abbreviations were used for ^1^H NMR spectra to indicate the signal multiplicity: s (singlet), d (doublet), dd (doublet of doublet), and m (multiplet). HRMS were determined with Agilent QTOF mass spectrometer 6540 series instrument using ESI technique.

### General procedure for the synthesis of 3a–j

To a stirred solution of appropriately substituted benzaldehydes **1a–j** (1 mmol) in ethanol (3 ml) was added 0.5 ml of acetone (**2**) and 15% aqueous NaOH (1 ml) solution at 0 °C. The reaction was allowed to stir at room temperature till it was completed. The reaction mixture was evaporated to dryness, extracted twice with ethyl acetate, and the combined organic layers were dried over anhydrous Na_2_SO_4_ and concentrated under reduced pressure. The crude product was purified by column chromatography (Silica gel, 60–120 mesh, 9:1 hexane/ethyl acetate) to obtain the desired chalcones **3a–j** in good to very good yields.

### General procedure for the synthesis of 5a–j

To a stirred solution of chalcone **3a–j** (0.5 mmol) in ethanol (3 ml) was added 15% aqueous NaOH (1 ml) solution and aldehyde **4** (0.5 mmol) at 0 °C. The resulting solution was stirred at room temperature till the complete consumption of starting materials was observed. The reaction mixture was evaporated to dryness, extracted twice with ethyl acetate, and the combined organic layers were dried over anhydrous Na_2_SO_4_ and concentrated under reduced pressure. The crude mass was purified by recrystallization in ethanol or ethyl acetate to give the pure product **5a–j** in yields of 35–45%.

### 4-((1E,4E)-5-(4-isopropoxyphenyl)-3-oxopenta-1,4-dien-1-yl)benzenesulfonamide (5a)

Yellow solid, yield 42%; mp: 163–165 °C; IR (KBr, cm^−1^): *ν*_max_ 3309.1, 3249.8, 2975.3, 2937.2, 1666.6, 1622.4, 1600.6, 1337.7, 1158.0, 1095.2; ^1^H NMR (500 MHz, DMSO-d_6_): *δ* 7.98 (d, *J* = 8.5 Hz, 2H), 7.88 (d, *J* = 8.4 Hz, 2H), 7.83–7.72 (m, 4H), 7.50–7.41 (m, 3H), 7.20 (d, *J* = 16.0 Hz, 1H), 7.01 (d, *J* = 8.8 Hz, 2H), 4.76–4.69 (m, 1H), 1.30 (d, *J* = 6.0 Hz, 6H); ^13^C NMR (125 MHz, DMSO-d_6_): *δ* 189.1, 156.3, 144.2, 143.8, 138.5, 131.0, 130.0, 129.2, 126.6, 125.9, 125.2, 123.7, 116.3, 69.9, 22.3, 22.2; HRMS (ESI): *m*/*z* Calcd for C_20_H_22_NO_4_S 372.1264, found 372.1262 [M + H]^+^.


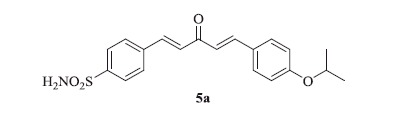


### 4-((1E,4E)-5-(4-methoxyphenyl)-3-oxopenta-1,4-dien-1-yl)benzenesulfonamide (5b)

Yellow solid, yield 39%; mp: 192–193 °C; IR (KBr, cm^−1^): *ν*_max_ 3356.5, 3289.2, 3071.1, 2934.3, 2838.6, 1645.3, 1597.0, 1331.3, 1161.3, 1094.7; ^1^H NMR (500 MHz, DMSO-d_6_): *δ* 7.98 (d, *J* = 8.4 Hz, 2H), 7.89 (d, *J* = 8.5 Hz, 2H), 7.84–7.75 (m, 4H), 7.52–7.43 (m, 3H), 7.21 (d, *J* = 16.0 Hz, 1H), 7.05 (d, *J* = 8.8 Hz, 2H), 3.83 (s, 3H); ^13^C NMR (125 MHz, DMSO-d_6_): *δ* 188.7, 161.8, 145.5, 143.8, 140.8, 138.5, 130.9, 129.3, 128.2, 127.6, 126.6, 123.9, 115.0, 55.8; HRMS (ESI): *m*/*z* Calcd for C_18_H_18_NO_4_S 344.0951, found 344.0950 [M + H]^+^.
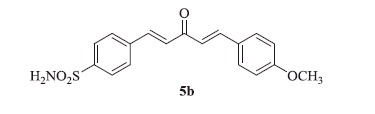


### 4-((1E,4E)-5-(2,4-dimethoxyphenyl)-3-oxopenta-1,4-dien-1-yl)benzenesulfonamide (5c)

Yellow solid, yield 40%; mp: 185–186 °C; IR (KBr, cm^−1^): *ν*_max_ 3321.3, 3258.6, 2939.6, 2838.9, 1644.6, 1600.6, 1328.7, 1159.9, 1098.9; ^1^H NMR (500 MHz, DMSO-d_6_): *δ* 7.97 (d, *J* = 8.6 Hz, 2H), 7.93 (s, 1H), 7.88 (d, *J* = 8.5 Hz, 3H), 7.79–7.74 (m, 3H), 7.39 (d, *J* = 16.1 Hz, 2H), 7.28 (d, *J* = 16.0 Hz, 2H), 3.91 (s, 3H), 3.85 (s, 3H); ^13^C NMR (125 MHz, DMSO-d_6_): *δ* 188.7, 163.6, 160.5, 145.5, 140.5, 138.6, 138.5, 130.8, 129.2, 129.0, 126.6, 123.5, 116.3, 106.9, 98.9, 56.3, 56.0; HRMS (ESI): *m*/*z* Calcd for C_19_H_20_NO_5_S 374.1057, found 374.1061 [M + H]^+^.
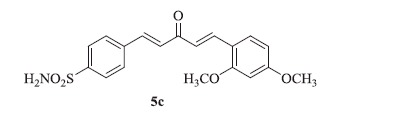


### 4-((1E,4E)-5-(2,5-dimethoxyphenyl)-3-oxopenta-1,4-dien-1-yl)benzenesulfonamide (5d)

Yellow solid, yield 38%; mp: 202–203 °C; IR (KBr, cm^−1^): *ν*_max_ 3364.1, 3258.2, 2943.2, 2836.0, 1654.0, 1597.9, 1326.5, 1158.5, 1096.1; ^1^H NMR (500 MHz, DMSO-d_6_): *δ* 7.98 (d, *J* = 8.6 Hz, 2H), 7.87 (d, *J* = 8.5 Hz, 2H), 7.83–7.77 (m, 3H), 7.40 (d, *J* = 16.0 Hz, 2H), 7.21 (d, *J* = 16.1 Hz, 1H), 7.08–7.04 (m, 3H), 3.85 (s, 3H), 3.79 (s, 3H); ^13^C NMR (125 MHz, DMSO-d_6_): *δ* 188.7, 153.7, 153.2, 144.3, 143.3, 141.3, 138.0, 129.3, 126.6, 123.5, 118.5, 113.6, 113.5, 113.2, 56.5, 56.1; HRMS (ESI): *m*/*z* Calcd for C_19_H_20_NO_5_S 374.1057, found 374.1060 [M + H]^+^.
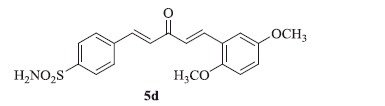


### 4-((1E,4E)-5-(3,4-dimethoxyphenyl)-3-oxopenta-1,4-dien-1-yl)benzenesulfonamide (5e)

Yellow solid, yield 40%; mp: 198–200 °C; IR (KBr, cm^−1^): *ν*_max_ 3321.7, 3252.6, 3091.3, 2940.1, 2839.5, 1619.5, 1596.1, 1330.1, 1158.8, 1095.2; ^1^H NMR (500 MHz, DMSO-d_6_): *δ* 7.99 (d, *J* = 8.4 Hz, 2H), 7.89 (d, *J* = 8.5 Hz, 2H), 7.84–7.74 (m, 4H), 7.51 (d, *J* = 16.0 Hz, 2H), 7.23 (d, *J* = 16.0 Hz, 2H), 7.06 (d, *J* = 8.4 Hz, 1H), 3.85 (s, 3H), 3.83 (s, 3H); ^13^C NMR (125 MHz, DMSO-d_6_): *δ* 188.7, 151.7, 149.5, 145.5, 144.3, 140.7, 138.5, 130.5, 129.2, 128.1, 127.8, 126.8, 126.6, 124.2, 123.9, 112.1, 111.0, 56.5, 56.0; HRMS (ESI): *m/z* Calcd for C_19_H_20_NO_5_S 374.1057, found 374.1055 [M + H]^+^.
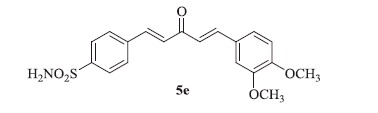


### 4-((1E,4E)-3-oxo-5-(2,3,4-trimethoxyphenyl)penta-1,4-dien-1-yl)benzenesulfonamide (5f)

Yellow solid, yield 35%; mp: 159–161 °C; IR (KBr, cm^−1^): *ν*_max_ 3305.9, 3241.6, 2974.1, 2948.4, 1652.1, 1592.9, 1334.4, 1156.5, 1090.8; ^1^H NMR (500 MHz, DMSO-d_6_): *δ* 7.98 (d, *J* = 8.4 Hz, 2H), 7.89–7.84 (m, 3H), 7.80 (d, *J* = 16.1 Hz, 1H), 7.63 (d, *J* = 8.9 Hz, 1H), 7.47 (s, 2H), 7.42–7.36 (m, 1H), 7.33 (d, *J* = 16.0 Hz, 1H), 6.95 (d, *J* = 8.9 Hz, 1H), 3.88 (s, 6H), 3.78 (s, 3H); ^13^C NMR (125 MHz, DMSO-d_6_): *δ* 188.7, 156.2, 153.5, 145.5, 142.3, 140.9, 138.4, 138.1, 129.3, 129.0, 126.6, 124.5, 123.9, 121.3, 109.0, 61.9, 60.9, 56.5; HRMS (ESI): *m/z* Calcd for C_20_H_22_NO_6_S 404.1162, found 404.1169 [M + H]^+^.
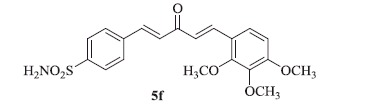


### 4-((1E,4E)-3-oxo-5-(2,4,6-trimethoxyphenyl)penta-1,4-dien-1-yl)benzenesulfonamide (5g)

Yellow solid, yield 43%; mp: 249–251 °C; IR (KBr, cm^−1^): *ν*_max_ 3304.9, 3253.2, 2969.1, 2951.9, 1616.8, 1591.7, 1335.2, 1157.2, 1090.1; ^1^H NMR (500 MHz, DMSO-d_6_): *δ* 8.07–8.01 (m, 1H), 7.97 (d, *J* = 8.4 Hz, 2H), 7.86 (d, *J* = 8.4 Hz, 2H), 7.64 (d, *J* = 16.0 Hz, 1H), 7.46–7.39 (m, 3H), 7.35 (d, *J* = 16.0 Hz, 1H), 6.32 (s, 2H), 3.91 (s, 6H), 3.86 (s, 3H); ^13^C NMR (125 MHz, DMSO-d_6_): *δ* 189.4, 161.8, 160.4, 145.1, 141.1, 137.3, 130.9, 129.2, 128.8, 126.6, 123.6, 115.9, 104.8, 90.1, 55.3, 54.5; HRMS (ESI): *m*/*z* Calcd for C_20_H_22_NO_6_S 404.1162, found 404.1166 [M + H]^+^.
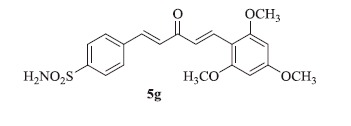


### 4-((1E,4E)-3-oxo-5-(3,4,5-trimethoxyphenyl)penta-1,4-dien-1-yl)benzenesulfonamide (5h)

Yellow solid, yield 45%; mp: 242–244 °C; IR (KBr, cm^−1^): *ν*_max_ 3301.8, 3240.4, 2940.2, 2834.7, 1639.5, 1590.5, 1319.8, 1157.0, 1093.9; ^1^H NMR (500 MHz, DMSO-d_6_): *δ* 8.11–7.99 (m, 2H), 7.97 (d, *J* = 8.5 Hz, 2H), 7.85 (d, *J* = 8.4 Hz, 2H), 7.63 (d, *J* = 15.9 Hz, 1H), 7.48–7.42 (m, 2H), 7.33 (d, *J* = 16.0 Hz, 1H), 6.93 (s, 2H), 3.91 (s, 6H), 3.86 (s, 3H); ^13^C NMR (125 MHz, DMSO-d_6_): *δ* 188.6, 153.2, 143.3, 142.7, 138.6, 130.5, 129.3, 127.8, 126.8, 123.7, 119.1, 104.1, 60.6, 56.5; HRMS (ESI): *m*/*z* Calcd for C_20_H_22_NO_6_S 404.1162, found 404.1164 [M + H]^+^.
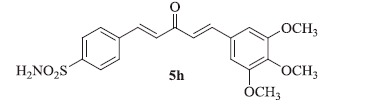


### 4-((1E,4E)-5-(4-chlorophenyl)-3-oxopenta-1,4-dien-1-yl)benzenesulfonamide (5i)

Yellow solid, yield 43%; mp: 205–206 °C; IR (KBr, cm^−1^): *ν*_max_ 3306.9, 3238.4, 2962.1, 2881.8, 1611.9, 1597.2, 1327.0, 1157.7, 659.6; ^1^H NMR (500 MHz, DMSO-d_6_): *δ* 7.99 (d, *J* = 8.2 Hz, 2H), 7.89 (d, *J* = 8.2 Hz, 2H), 7.86–7.81 (m, 4H), 7.55 (dd, *J* = 8.2, 3.3 Hz, 2H), 7.50–7.44 (m, 2H), 7.39 (dd, *J* = 16.0, 12.2 Hz, 2H); ^13^C NMR (125 MHz, DMSO-d_6_): *δ* 188.9, 145.6, 142.3, 142.0, 141.6, 138.3, 135.6, 135.5, 134.1, 130.7, 129.5, 129.3, 128.1, 126.7, 126.6; HRMS (ESI): *m*/*z* Calcd for C_17_H_15_ClNO_3_S 348.0456, found 348.0448 [M + H]^+^.
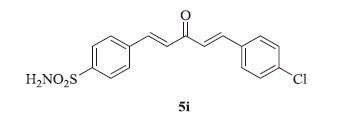


### 4-((1E,4E)-5-(2-fluoro-4-methoxyphenyl)-3-oxopenta-1,4-dien-1-yl)benzenesulfonamide (5j)

Yellow solid, yield 40%; mp: 167–169 °C; IR (KBr, cm^−1^): *ν*_max_ 3289.8, 3241.1, 2944.6, 2845.9, 1628.8, 1615.3, 1320.3, 1272.0, 1157.4, 1091.3; ^1^H NMR (500 MHz, DMSO-d_6_): *δ* 7.99 (d, *J* = 8.5 Hz, 2H), 7.92–7.86 (m, 3H), 7.81 (d, *J* = 16.1 Hz, 2H), 7.50–7.43 (m, 3H), 7.31 (d, *J* = 16.1 Hz, 1H), 6.99 (dd, *J* = 13.0, 2.5 Hz, 1H), 6.94–6.90 (m, 1H), 3.85 (s, 3H); ^13^C NMR (125 MHz, DMSO-d_6_): *δ* 188.7, 161.7, 158.6, 145.6, 143.3, 141.5, 138.5, 130.8, 128.3, 126.6, 123.7, 116.8, 111.2, 106.7, 55.7; HRMS (ESI): *m*/*z* Calcd for C_18_H_17_FNO_4_S 362.0857, found 362.0856 [M + H]^+^.
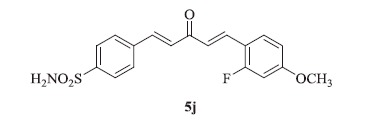


## CA inhibition assay

An Applied Photophysics stopped-flow instrument has been used for assaying the CA catalyzed CO_2_ hydration activity^29^. Phenol red (at a concentration of 0.2 mM) has been used as indicator, working at the absorbance maximum of 557 nm, with 20 mM Hepes (pH 7.5) as buffer, and 20 mM Na_2_SO_4_ (for maintaining constant the ionic strength), following the initial rates of the CA-catalysed CO_2_ hydration reaction for a period of 10–100 s. The CO_2_ concentrations ranged from 1.7 to 17 mM for the determination of the kinetic parameters and inhibition constants. For each inhibitor at least six traces of the initial 5–10% of the reaction have been used for determining the initial velocity. The uncatalysed rates were determined in the same manner and subtracted from the total observed rates. Stock solutions of inhibitor (0.1 mM) were prepared in distilled–deionized water and dilutions up to 0.01 nM were done thereafter with the assay buffer. Inhibitor and enzyme solutions were preincubated together for 15 min at room temperature prior to assay, in order to allow for the formation of the E–I complex. The inhibition constants were obtained by non-linear least-squares methods using PRISM 3 and the Cheng–Prusoff equation, as reported earlier^30–32^, and represent the mean from at least three different determinations. All CA isofoms were recombinant ones obtained in-house as reported earlier^30–32^.

## Results and discussion

### Chemistry

**Scheme 1.**
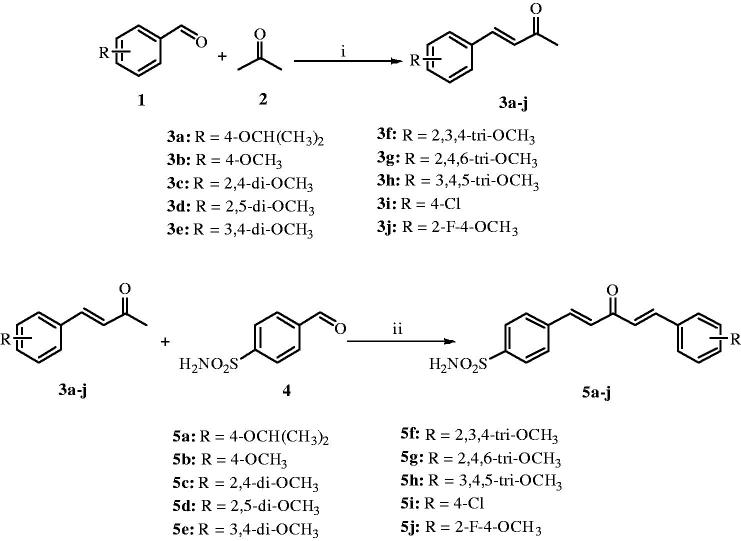
Synthesis of curcumin inspired sulfonamide derivatives (**5a–j**); Reagents and conditions: (i) 15% NaOH, ethanol, 0 °C – rt, 2–3 h, 72–85%; (ii) 15% NaOH, ethanol, 0 °C – rt, 1–2 h, 35–45%.

The synthetic route for the preparation of the target compounds, 4-((1*E*,4*E*)-3-oxo-5-phenylpenta-1,4-dien-1-yl)benzenesulfonamides (**5a–j**) is illustrated in Scheme 1. In the first step, chalcones (**3a–j**) were prepared by NaOH-catalyzed Claisen–Schmidt condensation of a variety of benzaldehydes (**1**) with acetone^28^. The corresponding chalcones were reacted with 4-sulfamoyl benzaldehyde (**4**) using 15% NaOH by Claisen–Schmidt condensation to afford the target compounds **5a–j** in good yields. All the synthesized compounds were purified by recrystallization in hot ethanol and well characterized by spectroscopic techniques such as ^1^H, ^13^C NMR, FT-IR and HRMS etc., which were in full accordance with the depicted structures. The ^1^H NMR spectrum of 4-((1*E*,4*E*)-5-(4-isopropoxyphenyl)-3-oxopenta-1,4-dien-1-yl)benzenesulfonamide (**5a**) showed a multiplet of isopropoxy (C–H) proton at δ 4.76–4.69, a doublet of two methyl group’s protons (total 6) of isopropoxy group at δ 1.30 and rest all protons appeared in the aromatic region in the range of δ 7.98–7.01. In the ^13^C NMR spectrum of **5a**, the carbonyl carbon appeared at δ 189.1 and the remaining aromatic carbons appeared in the range of δ 156.3–116.3. Nearly similar pattern was observed in ^1^H and ^13^C NMR spectra of all the other compounds (**5b–j)** of this series. In the FT-IR spectrum, bands at 3309.1, 3249.8, 1337.7 and 1158.0 cm^−1^ confirmed the presence of sulfonamide whereas a band at 1666.6 cm^−1^ confirmed the presence of ketone functionality in compound **5a**. The HRMS (ESI) of all the compounds showed an [M + H]^+^ peak equivalent to their molecular formulae.

### CA inhibition

All compounds **5a–j** were tested *in vitro* for their inhibitory activity against the physiologically relevant hCA isoforms I, II, IX, and XII by means of the stopped-flow carbon dioxide hydration assay^29^ and their activities were compared to the standard CA inhibitor (CAI) acetazolamide (**AAZ**) (Table 1).

**Table 1.** *In vitro* CA I, II, IX, and XII inhibition with compounds **5a–j** and acetazolamide (**AAZ**) as standard, by a stopped-flow, CO_2_ hydrase assay.^29^

	K_I_ (nM)^a^				Selectivity ratio^b^	
Compound	hCA I	hCA II	hCA IX	hCA XII	II/IX	II/XII
**5a**	417.4	7.9	2.3	6.1	3.43	1.29
**5b**	286.1	2.1	8.6	6.3	0.24	0.33
**5c**	848.1	4.7	7.5	7.4	0.62	0.63
**5d**	703.7	0.89	8.4	8.1	0.10	0.10
**5e**	294.8	0.75	2.3	52.2	0.32	0.01
**5f**	191.8	3.0	2.4	71.8	1.25	0.04
**5g**	904.2	0.87	2.4	7.4	0.36	0.11
**5h**	594.6	8.8	7.8	25.0	1.12	0.35
**5i**	372.6	8.1	87.3	31.1	0.09	0.26
**5j**	246.2	8.2	61.0	68.0	0.13	0.12
**AAZ**	250	12.1	25.8	5.7	0.46	2.12

a Mean from 3 different assays, by a stopped flow technique (errors were in the range of ±5–10% of the reported values).

b Selectivity as determined by the ratio of K_i_s for hCA isozyme relative to hCA IX and hCA XII.

We have investigated the novel series of sulfonamide derivatives for their interaction with four hCAs of pharmacologic interest (i.e. isoforms hCA I, II, IX, and XII), using a period of 15 min of incubation of the enzyme and inhibitor solutions^7^^,^^8^^,^^15^. The following structure–activity-relationship (SAR) may be noted regarding the inhibition data of Table 1:

1. Against the slow cytosolic isoform hCA I, almost all tested sulfonamide derivatives exhibited moderate inhibitory activity with K_I_ in the range 191.8–904.2 nM. Methoxy substituents on the benzene ring showed an important role for the modulation of inhibition. Among them, compound **5f** incorporating three MeO moieties in the 2, 3, 4 positions on the ring, prove to possess the highest inhibitory potency with a K_I_ of 191.8 nM.
2. hCA II, the dominant physiologic isoform, was effectively inhibited by all compounds here considered, in the low nanomolar range with K_I_s of 0.75–8.8 nM. Also for this isoform, the presence and position of methoxy groups in the chalcone synthon seems to play a crucial role for the inhibition potency. Among compounds with two OCH_3_ groups, derivative **5c** showed the “worst” inhibition profile, with a 5 times decreased activity compared to **5d** (4.7 nM and 0.89 nM), the two compounds only differing by the position of the two methoxy moieties in the second aryl functionality, the one coming from the chalcone. In addition, the position of OCH_3_ groups for compounds **5f–h**, with three substituents, plays an important role in modulating the activity. However, all these compounds were highly effective hCA II inhibitors (all of them better than AAZ), with a very small variation of the K_I_s, and more detailed structure activity relationship is impossible to be delineated at this pot for a relatively small group of derivatives.
3. hCA IX, the tumor-associated isoform, was inhibited by almost all compounds reported here, in the low nanomolar range (K_I_ 2.3–8.6 nM) except for compounds **5i** and **5j** which were active in the medium nanomolar range (K_I_ 61–87.3 nM). For this transmembrane isoform, the methoxy groups present on the scaffold did not influence significantly the inhibition profile. The interesting case for hCA IX inhibition was the presence of halogens in compounds **5i** and **5j**, that decreased the inhibition potency near 10 times compared to the other sulphonamides investigated here, which were devoid of such moieties.
4. The last membrane isoform here considered here, hCA XII, was inhibited by compounds **5a–d** and **5g** in low nanomolar range (K_I_ 6.1–7.4 nM). On the other hand, compounds **5e–f** and **5h–j** inhibited this isoform in medium nanomolar range (K_I_ 31.1–71.8 nM). In analogy with hCA IX, the methoxy groups in the scaffold did not influence significantly the potency of inhibition.

## Conclusions

In summary, a series of curcumin inspired sulfonamide derivatives (**5a–j**) were synthesized from chalcones and 4-sulfamoyl benzaldehyde by using Claisen–Schmidt condensation. The new sulfonamides were evaluated as inhibitors of four such isoforms, i.e. hCA I, II, IX, and XII. Interesting inhibitory activities were observed against almost all these isoforms as follows. These analogues inhibited hCA I (involved in some eye diseases) moderately with K_I_s in the range of 191.8–904.2 nM, hCA II (an antiglaucoma drug target) very potently with K_I_s in the range of 0.75–8.8 nM, hCA IX (an isoform involved in cancer) significantly with K_I_s in the range of 2.3–87.3 nM and hCA XII (antiglaucoma and anticancer drug target) with K_I_s in the range of 6.1–71.8 nM. Interestingly, **5d** was found to be almost 9 times more selective against hCA II isoform (K_I_ = 0.89 nM) over hCA IX and hCA XII isoforms and **5e** was nearly 3 and 70 times more selective against hCA II isoform (K_I_ = 0.75 nM) over hCA IX and hCA XII isoforms respectively.
